# Supplementary material for: AAV-Txnip prolongs cone survival and vision in mouse models of retinitis pigmentosa
Source: eLife. 2021 Apr 13;10:e66240. doi: 10.7554/eLife.66240 (PMC8081528; doi:10.7554/eLife.66240)
Supplement: Figure 1—source data 1. — Best1: retinal pigmented epithelium-specific promoter; SynPVI, SynP136, red opsin (RedO), RO1.7: various cone-specific promoters; N/A: not applicable; –: not performed; Pos: positive for cone rescue; Neg: negative for cone rescue. [file elife-66240-fig1-data1.docx]

**Figure 1—source data 1: AAV8 vectors used in this study.**

| **Inserts** | **Insert species** | **Promoter** | **Intron** | **WPRE** | **poly(A)** | **ITR-to-ITR size** | **Digestion (XmaI, ITR)** | **Partial seq (ligation site)** | **Complete plasmid seq** | **AAV genome seq** | **Effi-cacy** | **Raw data** |
| --- | --- | --- | --- | --- | --- | --- | --- | --- | --- | --- | --- | --- |
| **H2BGFP** | N/A | RedO (*default*) | N/A | WPRE | Bovine GH | 4.4 kb | Correct | Correct | - | - | N/A | - |
| **H2BGFP** | N/A | SynP136 | N/A | WPRE3 | SV40-Late | 3.9 kb | Correct | Correct | - | - | N/A | - |
| **mitoRFP** | N/A | SynP136 | N/A | WPRE3 | SV40-Late | 3.5 kb | Correct | Correct | Correct | - | N/A | - |
| **Txnip** | Mouse | RedO (*default*) | N/A | WPRE | Bovine GH | 4.5kb | Correct | Correct | Correct | - | Pos | Fig.1 |
| **Txnip** | Mouse | SynPVI | N/A | WPRE3 | SV40-Late | 2.4kb | Correct | Correct | - | - | Pos | Fig.1S1 |
| **Txnip** | Mouse | Best1 | SV40 | WPRE | Bovine GH | 3.1 kb | Correct | Correct | - | - | Neg | Fig.7S1 |
| **Txnip.C247S** | Mouse | RedO | N/A | WPRE | Bovine GH | 4.5kb | Correct | Correct | Correct | Correct | Pos | Fig.2 |
| **Txnip.S308A** | Mouse | RedO | N/A | WPRE | Bovine GH | 4.5kb | Correct | Correct | Correct | Correct | Neg | Fig.2 |
| **Txnip.C247S.S308A** | Mouse | RedO | N/A | WPRE | Bovine GH | 4.5kb | Correct | Correct | Correct | - | Neg | Fig.2 |
| **Txnip.C247S.**  **LL351&352AA** | Mouse | RedO | N/A | WPRE | Bovine GH | 4.5kb | Correct | Correct | Correct | - | Pos | Fig.2 |
| **Txnip.C247S.**  **LL351&352AA** | Mouse | Best1 | SV40 | WPRE | Bovine GH | 3.1 kb | Correct | Correct | Correct | - | Pos | Fig.7 |
| **GFP-Txnip** | Mouse | RO1.7 | N/A | WPRE3 | SV40-Late | 4.4 kb | Correct | Correct | Correct | - | N/A | - |
| **Nrf2** | Human | CMV | human β-globin | N/A | SV40 | 3.7 kb | - | - | - | - | Pos | Fig.1S1 |
| **Nrf2** | Human | SynPVI | N/A | WPRE3 | SV40-Late | 3.1 kb | Correct | Correct | Correct | - | Neg | Fig.8S1 |
| **Nrf2** | Human | Best1 | SV40 | WPRE | Bovine GH | 3.8 kb | Correct | Correct | - | - | Neg | Fig.8 |
| **Cx3cl1** | Mouse | Best1 | SV40 | WPRE | Bovine GH | 3.0 kb | - | Correct | Correct | - | Pos | Fig.7S1 |
| **Cx3cl1** | Mouse | RedO | N/A | WPRE | Bovine GH | 4.3 kb | - | Correct | - | - | Neg | Fig.8S1 |
| **Tgfb1** | Mouse | RedO | N/A | WPRE | Bovine GH | 4.4 kb | - | Correct | - | - | Pos | Fig.8 |
| **dnHIF1α** | Mouse | RO1.7 | N/A | WPRE3 | SV40-Late | 3.6 kb | Correct | Correct | Correct | - | Pos | Fig.7 |
| **dnHIF1α** | Mouse | Best1 | SV40 | WPRE | Bovine GH | 3.1 kb | Correct | Correct | Correct | - | Neg | Fig.7S1 |
| **Hif1a** | Mouse | SynPVI | N/A | WPRE3 | SV40-Late | 3.8 kb | Correct | Correct | Correct | - | Neg | Fig.7 |
| **Hk1** | Human | SynPVI | N/A | WPRE3 | SV40-Late | 4.0 kb | Correct | Correct | - | - | Neg | Fig.1S1 |
| **Hk2** | Mouse | SynPVI | N/A | WPRE3 | SV40-Late | 4.0 kb | Correct | Correct | - | - | Neg | Fig.1S1 |
| **Pfkm** | Human | SynPVI | N/A | WPRE3 | SV40-Late | 3.6 kb | Correct | Correct | - | - | Neg | Fig.1S1 |
| **Pkm1** | Human | SynPVI | N/A | WPRE3 | SV40-Late | 2.8 kb | Correct | Correct | - | - | Neg | Fig.1S1 |
| **Pkm2** | Human | SynPVI | N/A | WPRE3 | SV40-Late | 2.8 kb | Correct | Correct | - | - | Neg | Fig.1S1 |
| **Ldha** | Mouse | RO1.7 | N/A | WPRE3 | SV40-Late | 3.6 kb | Correct | Correct | Correct | - | Neg | Fig.1S1 |
| **Ldhb** | Mouse | RedO | N/A | WPRE | Bovine GH | 4.3 kb | Correct | Correct | Correct | - | Neg | Fig.8S1 |
| **Ldhb-3xFLAG** | Mouse | RO1.7 | N/A | WPRE3 | SV40-Late | 3.6 kb | Correct | Correct | Correct | - | N/A | - |
| **Slc2a1** | Mouse | RO1.7 | N/A | WPRE3 | SV40-Late | 4.0 kb | Correct | Correct | Correct | - | Neg | Fig.1S1 |
| **Bsg1** | Mouse | RedO | N/A | WPRE | Bovine GH | 4.4 kb | Correct | Correct | - | - | Neg | Fig.1S1 |
| **RdCVF** | Mouse | RedO | N/A | WPRE | Bovine GH | 3.6 kb | Correct | Correct | - | - | Neg | Fig.1S1 |
| **RdCVF** | Mouse | Best1 | SV40 | WPRE | Bovine GH | 2.3 kb | Correct | Correct | - | - | Neg | Fig.7S1 |
| **Cpt1a** | Mouse | RedO | N/A | WPRE | Bovine GH | 5.6 kb | Correct | Correct | Correct | - | Neg | Fig.1S1 |
| **Oxct1** | Mouse | RedO | N/A | WPRE | Bovine GH | 4.8 kb | Correct | Correct | Correct | - | Neg | Fig.1S1 |
| **Mpc1** | Mouse | RO1.7 | N/A | WPRE3 | SV40-Late | 3.0 kb | Correct | Correct | - | - | Neg | Fig.7S1 |
| **Mpc2** | Mouse | RO1.7 | N/A | WPRE3 | SV40-Late | 2.9 kb | Correct | Correct | - | - | Neg | Fig.7S1 |
| **Vegf164** | Mouse | RO1.7 | N/A | WPRE3 | SV40-Late | 3.1 kb | Correct | Correct | Correct | - | Neg | Fig.7S1 |
|  |  |  |  |  |  |  |  |  |  |  |  |  |
| **PercevalHR** | N/A | RO1.7 | N/A | WPRE3 | SV40-Late | 4.2 kb | Correct | Correct | Correct | - | N/A | - |
| **iGlucoSnFR** | N/A | SynPVI | N/A | WPRE3 | SV40-Late | 4.0 kb | Correct | Correct | Correct | - | N/A | - |
| **pHRed** | N/A | SynP136 | N/A | WPRE3 | SV40-Late | 3.5 kb | Correct | Correct | Correct | - | N/A | - |
|  |  |  |  |  |  |  |  |  |  |  |  |  |
| **siNC** | N/A | RedO | N/A | WPRE | Bovine GH | 3.3 kb | Correct | Correct | Correct | - | Neg | Fig.3 |
| **siLdhb^(#2)^ (default)** | Mouse | RedO | N/A | WPRE | Bovine GH | 3.3 kb | Correct | Correct | Correct | - | Neg | Fig.3 |
| **siLdhb^(#1)^** | Mouse | RedO | N/A | WPRE | Bovine GH | 3.3 kb | Correct | Correct | Correct | - | - | - |
| **siLdhb^(#3)^** | Mouse | RedO | N/A | WPRE | Bovine GH | 3.3 kb | Correct | Correct | Correct | - | - | - |
| **siOxct1** | Mouse | RedO | N/A | WPRE | Bovine GH | 3.3 kb | Correct | Correct | Correct | - | Neg | Fig.3 |
| **siCpt1a** | Mouse | RedO | N/A | WPRE | Bovine GH | 3.3 kb | Correct | Correct | Correct | - | Neg | Fig.3 |
| **siSlc2a1** | Mouse | RedO | N/A | WPRE | Bovine GH | 3.3 kb | Correct | Correct | Correct | - | Neg | Fig.2S1 |

***Best1:*** *RPE-specific promoter.* ***SynPVI, SynP136, RedO, RO1.7:*** *various cone-specific promoters.* ***N/A:*** *not applicable.* ***- :*** *not performed.* ***Pos:*** *positive for cone rescue.* ***Neg:*** *Negative for cone rescue.*
