# Supplementary material for: AAV-Txnip prolongs cone survival and vision in mouse models of retinitis pigmentosa
Source: eLife. 2021 Apr 13;10:e66240. doi: 10.7554/eLife.66240 (PMC8081528; doi:10.7554/eLife.66240)
Supplement: Figure 5—source data 1. [file elife-66240-fig5-data1.docx]

**Figure 5—source data 1: Differentially expressed genes in cones infected by AAV8-RedO-Txnip (1 x 10^9^ vg/eye plus AAV8-SynP136-H2BGFP, 1 x 10^9^ vg/eye) vs. control (AAV8-SynP136-H2BGFP, 1 x 10^9^ vg/eye) in common between two RP strains (*rd1* and *Rho*^-/-^).**

|  | **P90 *Rho^-/-^*** | | | | **P21 *rd1*** | | | |
| --- | --- | --- | --- | --- | --- | --- | --- | --- |
| **MGI**  **symbol** | **Base**  **Mean** | **log2Fold Change** | **log2Fold**  **SE** | **Adjusted**  **p-value** | **Base**  **Mean** | **log2Fold Change** | **log2Fold**  **SE** | **Adjusted**  **p-value** |
| **Txnip** | 1324.3 | 10.211 | 0.516 | 4.78E-83 | 582.4 | 9.325 | 0.775 | 2.17E-29 |
| **mt-Cytb** | 13643.7 | 1.248 | 0.257 | 0.00079349 | 5651.4 | 0.384 | 0.082 | 0.00014272 |
| **mt-Nd4** | 3118.0 | 1.195 | 0.212 | 3.52E-05 | 1504.6 | 0.392 | 0.076 | 1.76E-05 |
| **Vax2os** | 67.2 | 1.028 | 0.323 | 0.04091883 | 33.6 | 0.932 | 0.321 | 0.04383248 |
| **mt-Co1** | 9876.4 | 0.808 | 0.189 | 0.00393386 | 5235.1 | 0.388 | 0.075 | 1.7625E-05 |
| **Rom1** | 5040.6 | 0.748 | 0.190 | 0.00789643 | 4432.7 | 0.164 | 0.056 | 0.04435279 |
| **Cd63** | 488.9 | 0.717 | 0.233 | 0.04957176 | 249.8 | 0.370 | 0.110 | 0.01303008 |
| **Ftl1** | 799.9 | 0.657 | 0.212 | 0.04701416 | 934.6 | 0.252 | 0.081 | 0.02671713 |
| **Utp14b** | 51.1 | -2.486 | 0.586 | 0.00406139 | 60.1 | -1.679 | 0.246 | 2.9705E-09 |
| **Slc9a7** | 52.9 | -1.993 | 0.494 | 0.00646284 | 45.3 | -1.047 | 0.278 | 0.00408081 |
| **Megf9** | 369.0 | -1.752 | 0.529 | 0.03204053 | 417.7 | -0.666 | 0.173 | 0.00313404 |
| **Mgat2** | 33.4 | -1.572 | 0.434 | 0.01699743 | 36.7 | -1.343 | 0.343 | 0.00251377 |
| **Rnf168** | 40.0 | -1.508 | 0.476 | 0.04171633 | 45.7 | -0.970 | 0.275 | 0.00857761 |
| **Mid1** | 60.9 | -1.478 | 0.395 | 0.0126336 | 213.7 | -0.995 | 0.198 | 3.5144E-05 |
| **Ptprn2** | 333.4 | -1.461 | 0.358 | 0.00598384 | 45.8 | -0.994 | 0.309 | 0.01998408 |
| **Ankle2** | 115.9 | -1.429 | 0.350 | 0.00598384 | 99.1 | -0.605 | 0.207 | 0.04246472 |
| **Ccny** | 71.1 | -1.274 | 0.379 | 0.02959143 | 74.7 | -1.034 | 0.258 | 0.00181454 |
| **Galnt13** | 361.3 | -1.244 | 0.341 | 0.0159734 | 504.9 | -0.371 | 0.113 | 0.01655375 |
| **Ablim1** | 135.7 | -1.172 | 0.296 | 0.00760665 | 158.7 | -0.798 | 0.151 | 1.1301E-05 |
| **Acsl3** | 460.5 | -1.075 | 0.309 | 0.0236877 | 703.9 | -1.467 | 0.153 | 3.0866E-18 |
| **Ube3a** | 161.2 | -1.027 | 0.303 | 0.02803579 | 209.5 | -0.688 | 0.187 | 0.00513769 |
| **Socs5** | 358.6 | -0.820 | 0.256 | 0.03984866 | 337.8 | -0.811 | 0.126 | 2.8137E-08 |
| **Heg1** | 1328.6 | -0.795 | 0.209 | 0.01057592 | 1062.7 | -0.378 | 0.067 | 1.6244E-06 |
| **Cand1** | 323.4 | -0.744 | 0.231 | 0.03864972 | 283.1 | -0.597 | 0.187 | 0.02106014 |
| **Gprasp1** | 509.6 | -0.534 | 0.163 | 0.03426564 | 356.1 | -0.416 | 0.145 | 0.04690377 |
