# Supplementary material for: AAV-Txnip prolongs cone survival and vision in mouse models of retinitis pigmentosa
Source: eLife. 2021 Apr 13;10:e66240. doi: 10.7554/eLife.66240 (PMC8081528; doi:10.7554/eLife.66240)
Supplement: Figure 5—source data 2. [file elife-66240-fig5-data2.docx]

**Figure 5—source data 2: Differentially expressed gene(s) in cones infected by AAV8-RedO-Txnip (1 x 10^9^ vg/eye plus AAV8-SynP136-H2BGFP, 1 x 10^9^ vg/eye) vs. control (AAV8-SynP136-H2BGFP, 1 x 10^9^ vg/eye) in common between two WT strains (BALB/c and C57BL6/J).**

|  | **P35 C57BL/6J** | | | | **P21 BALB/c** | | | |
| --- | --- | --- | --- | --- | --- | --- | --- | --- |
| **MGI**  **symbol** | **Base**  **Mean** | **log2Fold Change** | **log2Fold**  **SE** | **Adjusted**  **p-value** | **Base**  **Mean** | **log2Fold Change** | **log2Fold**  **SE** | **Adjusted**  **p-value** |
| **Txnip** | 2500.3 | 12.780 | 1.064 | 1.16E-28 | 1355.8 | 11.721 | 0.594 | 1.12E-82 |
