# Supplementary material for: AAV-Txnip prolongs cone survival and vision in mouse models of retinitis pigmentosa
Source: eLife. 2021 Apr 13;10:e66240. doi: 10.7554/eLife.66240 (PMC8081528; doi:10.7554/eLife.66240)
Supplement: Figure 5—source data 3. — Data presented as mean ± SEM (n = sample size, i.e., number of retinas per condition). [file elife-66240-fig5-data3.docx]

**Figure 5— source data 3: Cone *Txnip* mRNA raw reads in the RNA-seq data (from 1,000 FACS cones per retina).**

| **Condition** | **Control** | **Txnip** |
| --- | --- | --- |
| **P21 *rd1*** | 3.5 ± 2.2 (n=6) | 1834.3 ± 36.3 (n=3) |
| **P90 *Rho*^-/-^** | 2.3 ± 1.7 (n=4) | 2767.3 ± 232.2 (n=4) |
| **P21 BALB/c** | 1.0 ± 0.5 (n=6) | 3116.8 ± 859.6 (n=6) |
| **P35 C57BL/6J** | 0.7 ± 0.3 (n=3) | 5882.7 ± 1884.4 (n=3) |

*Data presented as: Mean ± SEM (n = sample size, i.e. number of retinas per condition.)*
