# Supplementary material for: AAV-Txnip prolongs cone survival and vision in mouse models of retinitis pigmentosa
Source: eLife. 2021 Apr 13;10:e66240. doi: 10.7554/eLife.66240 (PMC8081528; doi:10.7554/eLife.66240)
Supplement: Figure 5—source data 4. — Data presented as mean ± SEM (n = 35 for all). No significant difference in these genes with or without Txnip in each strain. [file elife-66240-fig5-data4.docx]

**Figure 5—source data 4: Cone mRNA raw reads from RNA-seq of all 35 retinas used in the study.**

| **Gene** | **Raw reads** |
| --- | --- |
| ***Slc2a1*** | 278.8 ± 21.4 |
| ***Ldha*** | 6827.4 ± 511.8 |
| ***Ldhb*** | 296.0 ± 28.7 |

*Data presented as: Mean ± SEM (n=35 for all).*

*No significant difference in these gene with or without Txnip in each strain.*
